# Home-based vs center-based exercise on patient-reported and performance-based outcomes for knee osteoarthritis: a systematic review with meta-analysis

**DOI:** 10.3389/fpubh.2024.1360824

**Published:** 2024-03-14

**Authors:** Zhi-Yuan Zhang, Lu Huang, Lv Tian, Jiang Yi, Min Gao, Xiao-Qi Wang, Jun-Jie Jiang, Zhong-Liang Liu

**Affiliations:** ^1^Department of Rehabilitation Medicine, The Second Hospital of Jilin University, Chang Chun, China; ^2^School of Nursing, Jilin University, Chang Chun, China

**Keywords:** knee osteoarthritis, home based, exercise, systematic review, patient reported outcome measures

## Abstract

**Background:**

Home-based exercise (HBE) represents an alternative to increase the accessibility of rehabilitation programs and relieve the burden on the health care system for people with knee osteoarthritis.

**Objectives:**

To summarize for the first time the effectiveness of HBE as compared to center-based exercise (CBE), both with and without HBE, on patient-reported and performance-based outcomes in people with KOA.

**Methods:**

Searches were conducted on PubMed, Cochrane, Embase, Web of Science, and Scopus until March 10, 2023, without date or language restrictions. Randomized controlled trials investigating HBE versus CBE or HBE combined with CBE for people with KOA were eligible. The primary outcomes were patient-reported: pain, physical disability, and quality of life. The secondary outcomes were performance-based: walking ability, lower limb muscle strength, and balance function. Risk of bias was assessed with the Cochrane Risk of Bias tool and quality of evidence according to the GRADE.

**Results:**

Eleven trials involving 956 participants were included. There was no difference in short-term pain (SMD, 0.22 [95% CI, −0.04 to 0.47], *p* = 0.09; I^2^ = 0%), physical disability (SMD, 0.17 [95% CI, −0.19 to 0.54], *p* = 0.35; I^2^ = 0%), walking ability (SMD, −0.21 [95% CI, −0.64 to 0.22], *p* = 0.33; I^2^ = 35%) and lower limb muscle strength (SMD, −0.24 [95% CI, −0.88 to 0.41], *p* = 0.47; I^2^ = 69%) between HBE and CBE. HBE combined with CBE has better benefits compared with HBE alone in short-term pain (SMD, 0.89 [95% CI, 0.60 to 1.17], *p* < 0.001; I^2^ = 11%) and physical disability (SMD, 0.25 [95% CI, 0.00 to 0.50], *p* = 0.05; I^2^ = 0%).

**Conclusion:**

Based on limited evidence, HBE is as effective as CBE on short-term pain, physical disability, walking ability, and lower limb muscle strength in people with knee osteoarthritis. Furthermore, combining HBE with CBE may enhance the overall efficacy of the intervention.

**Systematic review registration:**

PROSPERO, CRD42023416548.

## 1 Introduction

Knee osteoarthritis (KOA) is a chronic degenerative condition that involves the entire joint, including bone, synovium, and capsule, with an estimated 240 million persons suffering symptomatic and activity-limiting OA worldwide (1). Nearly 30% of individuals over 45 years old have radiographic evidence of KOA, with about half suffering knee symptoms (2, 3). To minimize the rising social and personal costs of KOA, interventions that alleviate symptoms and reduce prevalence through primary and secondary prevention programs are essential.

In recent years, a shift from pharmacologic therapy to nonpharmacologic therapy has occurred due to the limited efficacy of the former and mounting evidence indicating that nonpharmacologic modalities have superior long-term symptom relief and can delay or prevent functional decline for KOA. Among nonpharmacologic therapy, both center-based exercise (CBE) and home-based exercise (HBE) have been found to reduce pain and improve function in people with KOA compared to no intervention or usual care (4–8). HBE refers to exercise that takes place in an informal and flexible setting, generally in patients’ homes (9, 10), which offers a sense of familiarity within one’s surroundings, providing comfort and accessibility while also reducing costs, environmental challenges, and travel time to a healthcare center (11–14). Therefore, HBE represents an alternative to increase the accessibility of exercise programs and relieve the burden on the health care system (12–15). Nevertheless, the comparative effectiveness between HBE and CBE for KOA is debated.

A succession of systematic reviews has demonstrated that HBE is almost as effective as CBE for multiple conditions, such as cardiovascular diseases (16–20), Parkinson’s disease (21), and old healthy people (11, 22, 23). A previous review (24) attempted to investigate the effects of mixed home-based rehabilitation in people with KOA, not the effects of HBE, and the results were inconclusive. In addition, several clinical trials have been designed to determine whether supplementing HBE with CBE enhances its efficacy, but the results have been mixed (25–27). Despite these findings, there is yet been no evidence-based clinical practice guideline promoting the use of home-based exercise for KOA, therefore a rigorous systematic review with meta-analysis of the current high-quality evidence is warranted.

This study aimed to summarize for the first time the evidence on the effectiveness of HBE compared with CBE and HBE combined with CBE on patient-reported and performance-based outcomes in people with knee osteoarthritis.

## 2 Methods

### 2.1 Data sources and searches

The review was reported according to the Preferred Reporting Items for Systematic Reviews and Meta-Analyzes (PRISMA) statement (28), and followed the methods described in the Cochrane Handbook for Systematic Reviews of Interventions, Version 6.3 (29). The protocol was registered in the International Prospective Register of Systematic Reviews (PROSPERO): CRD42023416548.

We searched PubMed, Cochrane, Embase, Web of Science, and Scopus until March 10, 2023, without date or language restrictions. The search string was built as follows: knee osteoarthritis, exercise, and home (see Supplementary Appendix S1 for the full search strategy). The electronic database search was supplemented by a manual search to identify potentially eligible records.

### 2.2 Study selection

Study selection was conducted by 2 authors using predetermined criteria independently. Disagreements were resolved by consensus after discussion with a third author. If the full manuscript could not be obtained, we contacted the author via email. Randomized controlled trials (RCTs) investigating HBE versus CBE or HBE combined with CBE for people with KOA were eligible. Participants were given an established diagnosis of KOA according to accepted criteria, regardless of age, gender, ethnicity, demography, and geography (30). The experimental intervention was HBE with all the exercise sessions being conducted at home. The control intervention was a supervised CBE with or without home-based exercise sessions, delivered in a hospital, outpatient department, private clinic, medical center, or community center. All interventions in the experimental and control groups were prescribed by a physical therapist or health professional. Excluding studies conducted on humans with non-KOA or conducted on animals. We determined and classified primary and secondary outcome measures in this review based on the international consensus on core outcome measures for phase III clinical trials of OA (31). The primary outcomes were self-reported measures, including pain, physical disability, and quality of life. The secondary outcomes were performance-based measures, including walking ability, lower limb muscle strength, and balance function.

### 2.3 Data extraction and quality assessment

Data from the included studies were independently extracted by 2 authors guided by Cochrane handbook (29). Any disagreement was resolved by discussion and a third author as required. The following descriptive data were extracted: Author, country of study, published year, participant characteristics (sample size, age, gender), intervention characteristics, and outcome measures at different follow-up times. The outcome measures were classified as patient-reported measures (1) pain (2), physical disability, and (3) quality of life; performance-based measures (4) walking ability (5) lower limb muscle strength, and (6) balance function. Where studies included more than one measurement scale, we extracted data from a scale that is highest on a suggested hierarchy (32, 33) (Supplementary Appendix S2). The short-term effect was defined as follow-up up to 3 months after baseline, and the long-term effect was defined as follow-up beyond 3 months after baseline. When multiple time points were available within the same follow-up period, the one closer to the endpoint of the intervention was used. The corresponding author of the relevant study was contacted to obtain missing data.

An assessment of the methodological quality of the primary articles was carried out by two reviewers independently, using the Cochrane risk of bias tool (version 2, ROB2) (34). This tool rates 7 potential sources of bias across 5 domains (randomization process, intended interventions, missing outcome data, measurement of the outcome, and selection of the reported result). Each trial was assessed against 5 bias domains to produce a summary risk-of-bias assessment score for each domain and overall (low risk, some concerns, or high risk of bias). Disagreements were resolved by discussion or adjudication. Results from these questions were graphed and assessed using an Excel RoB2 tool.

### 2.4 Data synthesis and analysis

The post-intervention was used to obtain the pooled estimate of the effect of the intervention, using a random effects model due to the expected heterogeneity between the studies. We calculated standardized mean differences (SMDs) and 95% confidence intervals (CIs) for continuous variables with the inverse variance method. When necessary, SDs were calculated using available data (eg, 95% CI or *p* value) following the Cochrane guidelines. The effect size was interpreted as small (0.2), moderate (0.5), or large (0.8). Statistical heterogeneity was assessed using the I^2^, with classification as low (I^2^ < 25%), moderate (I^2^ = 25–50%), substantial (I^2^ = 50–75%), and considerable (I^2^ > 75%) (29). A funnel plot would be used to evaluate publication bias if ≥10 studies were available for a given meta-analysis (35), but the number found did not reach this. Sensitivity analyzes were conducted for each outcome by excluding one study in each round. We used Review Manager (version 5.4.1) to perform all statistical analyzes. Data unamenable to meta-analysis were reported narratively.

The overall quality of evidence for each outcome was rated according to the Grading of Recommendations Assessment, Development, and Evaluation (GRADE) guidelines (36). Considering only RCTs were included, each outcome received a high certainty at the outset. Two reviewers assessed the quality of the evidence using the GRADE system, with potential disagreements resolved by discussion with a third reviewer. The GRADE downgrade details were presented in the Supplementary Appendix S3.

## 3 Results

### 3.1 Compliance with the registered protocol

To ensure a consistent comparison of primary studies and a robust conclusion, we categorized questions into two groups. The first group compared the efficacy between HBE and CBE in the control group, while the second group explored the efficacy between HBE and HBE combined with CBE. There were no other inconsistencies with the pre-registration protocol.

### 3.2 Study identification

**Figure 1:**
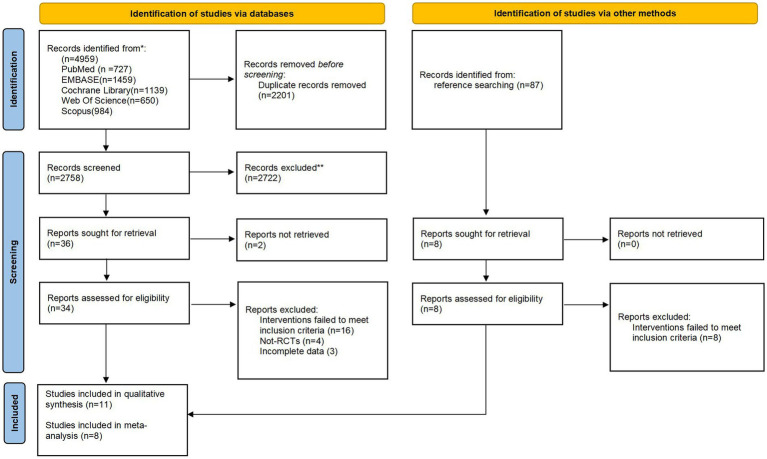
Flowchart of the study selection process.

The initial database searches identified 4,959 citations (Figure 1). After the removal of duplicates, 2,758 records remained. Based on the title and abstract, we excluded 2,722 records that did not meet the eligibility criteria. The remaining 34 full texts were identified and 24 were excluded. Of these excluded studies, 16 for wrong intervention, 4 for wrong study design, and 3 for incomplete data (Supplementary Appendix S4). No additional articles were identified through manual searching.

### 3.3 Study characteristics

Descriptive characteristics of the 11 included studies are detailed in Tables 1, 2. HBE was compared with CBE in 6 studies (37–42), and with HBE combined with CBE in 5 studies (25–27, 43, 44). Studies were published from 2004 to 2022, with two studies conducted in Turkey (26, 39), two in Australia (37, 38), two in United States (25, 44), two in Nigeria (40, 41), with the remainder in Iran (43), Thailand (42), and United Kingdom (27). HBE had periodic remote monitoring by telephone (38–41, 43, 44) or videoconference (37), whereas four trials with no monitoring (25–27, 42). Support materials included exercise video (43, 44), exercise instruction manual (25, 40, 41, 43), exercise logbook (25, 40–43), and automated reminders (44). Two trials in the CBE were group-based exercise (27, 39) and the remaining trials were one-to-one exercises. Interventions for HBE combined with CBE additionally consisted of manual therapy (25), device-supported exercise therapy (26), and multiple physical factors (43). The intervention during for the included trials ranged from 4 to 24 weeks (2-7/wk), and participants were followed up for 0 to 48 weeks.

**Table 1:** Characteristics of included studies: HBE compared with CBE.

Author (year), country	Sample characteristics N, Age, Female/male		Description of intervention	Description of control	Outcomes measures, Follow-up(weeks)
	Intervention group	Control group			
Barton (2022) Australia	22, 62 (8), 14/8	22, 64 (10), 12/10	Intervention contents: neuromuscular exercise, education program Frequency and duration: 60 min × 2/wk × 8 wk Monitor: video-conferencing platform Supporting materials: NA.	Intervention contents: neuromuscular exercise, education program Frequency and duration: 60 min × 2/wk × 8 wk Supervision: one-to-one, 100% Supporting materials: NA.	KOOS Total 12, 48 wk
Aily (2020) Australia	10, 53.1 (8.3), 5/5	10, 54.8 (8.3), 5/5	Intervention contents: periodized circuit training, with load progression Frequency and duration: 3/wk × 14 wk Monitor: periodic telephone calls. Supporting materials: exercise videos on a website, DVD or YouTube.	Intervention contents: periodized circuit training, with load progression Frequency and duration: 3/wk × 14 wk Supervision: one-to-one, 100% Supporting materials: NA.	VAS, WOMAC function, 40 m fast-paced walk test(sec), 30s chair stand test(n) 0 wks
Kuptniratsaikul (2019) Thailand	40, 61.7 (6.9), 37/3	40, 62.1 (6.4), 38/2	Intervention contents: various quadriceps exercise Frequency and duration: 30 min × 7/wk × 4 wk Monitor: no Supporting materials: instruction brochures, exercise logbook	Intervention contents: UTM exercise with moderate intensity (NRS 5–6/10), including warm up and cool down. Frequency and duration: 30 min × 3/wk × 4 wk Supervision: one-to-one, 100%	Pain score 6MWT (m) QS (kg) 0 wk
Kuru Çolak (2017) Turkey	23, 59 (21.5), 15/8	33, 60 (25.9), 24/9	Intervention contents: One face-to-face instruction exercise session, therapeutic isometric and isotonic exercises, simple balance exercises Frequency and duration: 3/wk × 6 wk Monitor: structured weekly phone call Supporting materials: NA	Intervention contents: therapeutic isometric and isotonic exercises, simple balance exercises Frequency and duration: 40–45 min × 3/wk × 6 wk Supervision: group-based, 100% Supporting materials: NA	VAS (after activity), Right QS(pounds), Right Hamstring muscle strength(pounds), 6MWT, Balance score 0 wk
Odole (2013, 2014) Nigeria	25, 56.04 (7.40), 11/14	25, 54.96 (7.81), 13/12	Intervention contents: standardized exercise program Frequency and duration: 3/wk × 6 wk Monitor: structured weekly phone call Supporting materials: exercise logbook, a copy guidance of the standardized exercise programs	Intervention contents: the same standardized exercise program Frequency and duration: 3/wk × 6 wk Supervision: one-to-one, 100% Supporting materials: NA	Physical health domain of WHOQoL, Pain-Ibadan Knee/Hip Osteoarthritis Outcome Measure, Physical function-Ibadan Knee/Hip Osteoarthritis Outcome Measure 2, 4, 6 wks

NA, not available; VAS, Visual analog pain scale; KOOS, Knee Injury and Osteoarthritis Outcome Score; WOMAC, Western Ontario and McMaster Universities Arthritis Index; TUG, Timed Up and Go test; 6MWT, Six-Minute Walk Test; ALF, Aggregate locomotor function; WHOQOL, World Health Organization Quality of Life; QS, quadriceps strength; HBE, home-based exercise; CBE, center-based exercise.

**Table 2:** Characteristics of included studies: HBE compared with HBE combined with CBE.

Author (year), country	Sample characteristics *N*, Age, Female/male		Description of intervention	Description of control	Outcomes measures, Follow-up (weeks)
	Intervention group	Control group			
Allen (2018) United States	142 (0 week) 114 (16wk follow-up), 112 (48wk follow-up), 65.3 (11.5), 98/44	140 (0 week) 130 (16wk follow-up), 129 (48wk follow-up), 65.7 (10.3), 100/40	Intervention contents: Tailored Exercises, Exercise Progression recommendations Frequency and duration: 3/wk × 16 wk Monitor: progress tracking Supporting materials: Video Display of Exercises (and photographs), automated Reminders	HBE: home-based exercise CBE: Intervention contents: evidence-based PT sessions, other recommended elements of care for knee OA; Frequency and duration: NA Supervision: up to 1 h × 8 sessions in 16wk, one-to-one Supporting materials: NA	WOMAC pain, WOMAC function, 30 s chair stand, TUG 0, 32 wks
Azma (2018) Iran	27, 55 (5.2), NA	27, 56 (5.1), NA	Intervention contents: strengthening, endurance, flexibility, and active range of motion exercises, HP Frequency and duration: 3/wk × 6 wk Monitor: weekly phone call Supporting materials: a pamphlet with exercise descriptions, an activity logbook	HBE: The same HBE as intervention group at home between sessions. CBE: Intervention contents: HP, TENS, and US; Frequency and duration: 3/wk × 6 wk Supervision: NA, one-to-one Supporting materials: NA	VAS, WOMAC Total, KOOS QoL 0, 4, 24 wks
Tunay (2010) Turkey	30, 54.4 (7.99), NA	30, 50.23 (9.07), NA	Intervention contents: proprioception, strengthening exercise, and cold compress. Frequency and duration: 5/wk × 6 wk Monitor: no Supporting materials: NA	HBE: the same as HBE group, including strengthening exercise and cold compress. CBE: Intervention contents: proprioceptive exercise training by “Monitored Rehabilitation Systems”; Frequency and duration: 5/wk × 6 wk Supervision: 50%, one-to-one	Left knee VAS activity, Right knee VAS activity, WOMAC Total, TUG 0 wk
McCarthy (2004) United Kingdom	86 (0 wek), 79 (24 wek), 71 (48 wek), 64.9 (9.7), NA	104 (0 wek), 103 (24 wek), 80 (48 wek), 64.5 (9.9), NA	Intervention contents: one face-to-face treatment sessions, individualized home-based exercise program, the advice and education Frequency and duration: 7/wk × 8 wk Monitor: no Supporting materials: NA	HBE: the same as HBE group CBE: Intervention contents: progressive resistance training, accelerated walking, and stretching and balance exercises, the advice and education; Frequency and duration: 45 min × 2/wk × 8 wk Supervision: 29%, group-based Supporting materials: NA	VAS, ALF Score, WOMAC function 0, 24, 48 wks
Deyle (2005) United States	60 (4 and 8 wek follow-up), 62.2 (9.2), 44/16	60 (4 and 8 wek follow-up), 64.0 (9.9), 37/23	Intervention contents: two detailed verbal and hands-on exercise instruction sessions, standardized knee exercise program Frequency and duration: 3-7/wk × 4 wk Monitor: no Supporting materials: A detailed exercise instruction handout and a home program adherence logbook	HBE: the same home exercise program each day that they were not treated in the clinic. CBE: Intervention contents: manual therapy, standardized knee exercise program; Frequency and duration: 8 sessions in 4 wk Supervision: 40%, one-to-one Supporting materials: NA	WOMAC-Total, 6MWT 0, 4 wks

HP, hot pack; TENS, transcutaneous electrical nerve stimulation; US, ultrasonography; NA, not available; VAS, Visual analog pain scale; KOOS, Knee Injury and Osteoarthritis Outcome Score; WOMAC, Western Ontario and McMaster Universities Arthritis Index; TUG, Timed Up and Go test; 6MWT, Six-Minute Walk Test; ALF, Aggregate locomotor function; PT, Physical Therapy; UTM, underwater treadmill exercise; WHOQOL, World Health Organization Quality of Life; HBE, home-based exercise; CBE, center-based exercise.

### 3.4 Risk of bias

Risk of bias assessment is provided in Supplementary Appendices S5, S6. The assessment resulted in some concerns of risk of bias in most trials (25, 27, 37–41, 43), high risk of bias in 1 trial (26), and low risk of bias in 2 trials (42, 44). Due to insufficient information, most trials were scored unclear or high risk in the randomization process or/and deviations from the intended interventions. Two-fives of the trials (26, 27, 37, 38) were unclear or at high risk of outcome measurement, mainly because of the lack of assessor blinding. Except for two trials (42, 44), most of the other trials lacked a pre-registered protocol, which might result in selective reporting bias risk.

### 3.5 Effect of HBE compared with CBE

Six studies involving 240 participants contributed data on the effectiveness of HBE versus CBE. The follow-up periods ranged from 0 to 12 months. The GRADE evidence profile comparing the efficacy of HBE and CBE for primary outcomes and secondary outcomes is reported in Supplementary Appendices S7, S8.

#### 3.5.1 Primary outcomes

**Figure 2:**
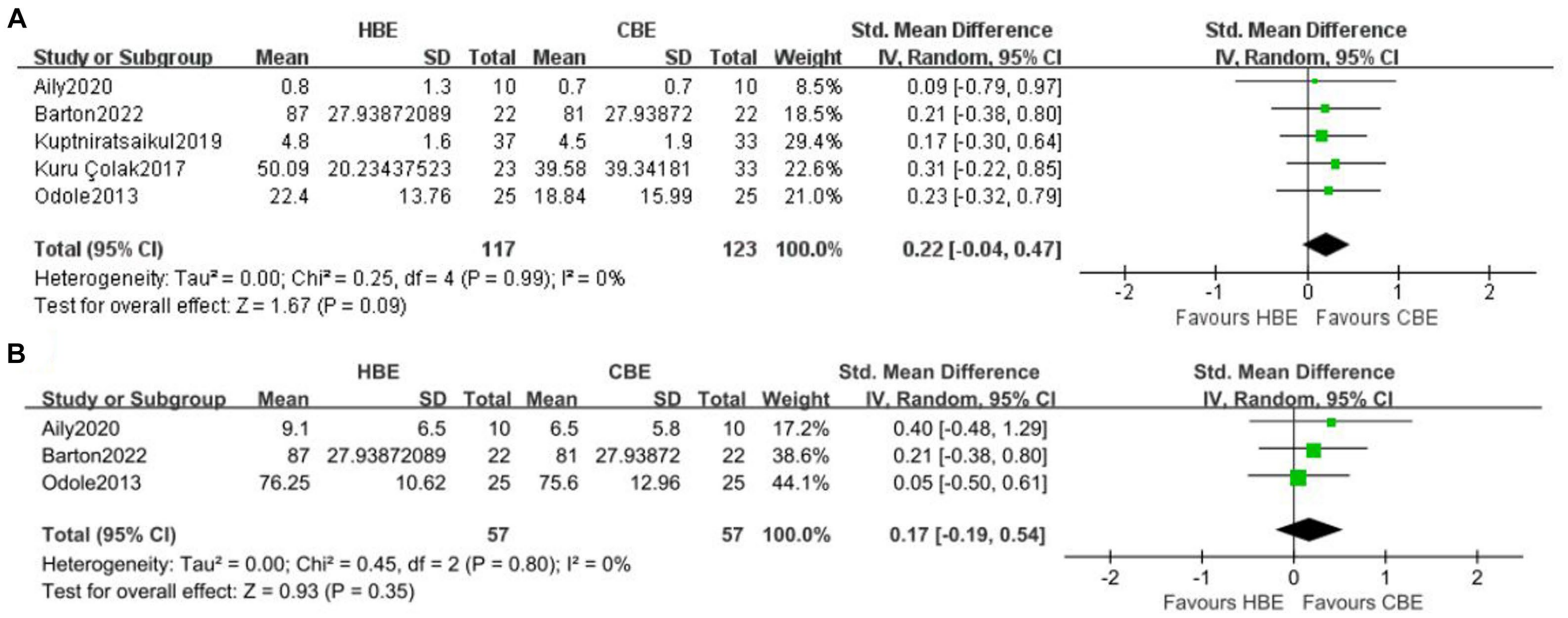
Meta-analysis of short-term effects of HBE versus CBE on **(A)** pain and **(B)** physical disability.

Meta-analysis of 5 studies showed the decrease of short-term pain in CBE was more than that in HBE (SMD, 0.22 [95% CI, −0.04 to 0.47], *p* = 0.09; I^2^ = 0%, GRADE moderate) (Figure 2A). However, the confidence intervals for the SMD were also consistent with the possibility that the difference is close to zero. Meta-analysis of 3 studies showed no between-groups difference for physical disability at short-term (SMD, 0.17 [95% CI, −0.19 to 0.54], *p* = 0.35; I^2^ = 0%, GRADE moderate) (Figure 2B). Only one study (40) with 50 participants reported patient-reported quality of life at short-term, and no difference was found. One study (37) reported 12-month follow-up of KOOS_4_ and showed no between-group statistical difference.

#### 3.5.2 Secondary outcomes

**Figure 3:**
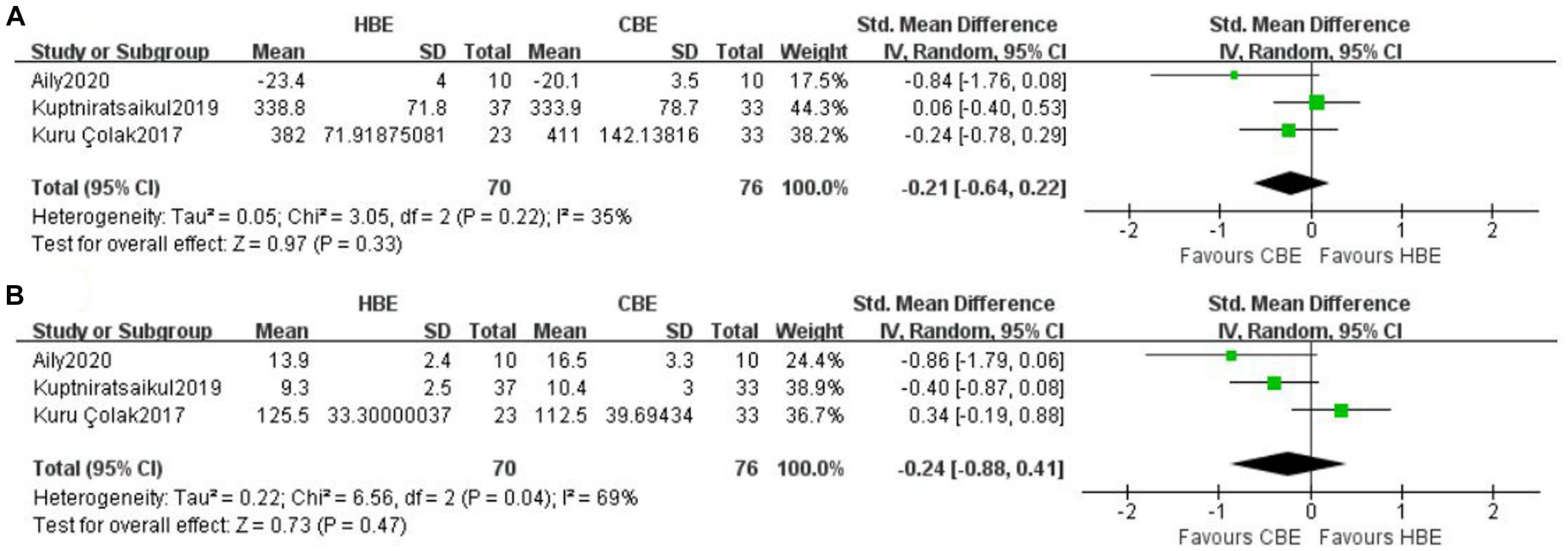
Meta-analysis of short-term effects of HBE versus CBE on **(A)** walking ability and **(B)** lower limb muscle strength.

Meta-analysis of 3 studies showed no between-groups difference for walking ability at short-term (SMD, −0.21 [95% CI, −0.64 to 0.22], *p* = 0.33; I^2^ = 35%, GRADE moderate) (Figure 3A). Meta-analysis of 3 studies showed no difference for lower limb muscle strength at short-term between HBE and CBE (SMD, −0.24 [95% CI, −0.88 to 0.41], *p* = 0.47; I^2^ = 69%, GRADE low) (Figure 3B). Only one study (39) reported the balance function at short-term, displaying no difference between HBE (*n* = 23) and CBE (*n* = 33). No studies reported long-term performance-based outcome measures comparing HBE and CBE.

### 3.6 Effect of HBE compared with HBE combined with CBE

Five studies investigated the effectiveness of HBE versus HBE combined with CBE, with two studies (26, 27) (250 participants) included in meta-analyzes. Three other studies (25, 43, 44) failed to include meta-analysis due to inconsistent interventions, which contained additional center-based interventions besides supervised in-person exercise in their CBEs, potentially confounding the effects of exercise therapy. The follow-up periods ranged from 0 to 12 months. The GRADE evidence profile comparing the efficacy of HBE and HBE combined with CBE for primary outcomes is reported in Supplementary Appendix S9.

#### 3.6.1 Primary outcomes

**Figure 4:**
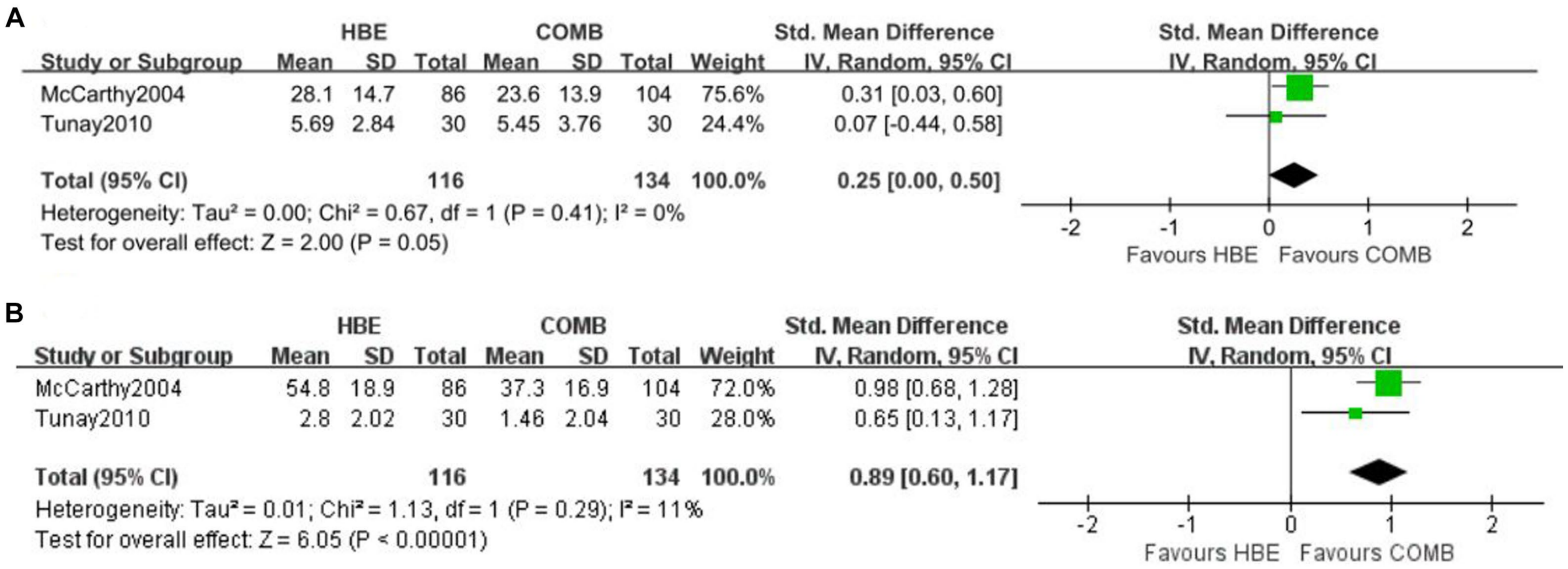
Meta-analysis of short-term effects of HBE versus HBE combined with CBE on **(A)** pain and **(B)** physical disability.

Meta-analysis of 2 studies showed HBE was inferior to HBE combined with CBE for pain at short-term (SMD, 0.89 [95% CI, 0.60 to 1.17], *p* = 0.001; I^2^ = 11%, GRADE moderate) (Figure 4A). Meta-analysis of 2 studies showed HBE was inferior to HBE combined with CBE for physical disability at short-term (SMD, 0.25 [95% CI, 0.00 to 0.50], *p* = 0.05; I^2^ = 0%, GRADE moderate) (Figure 4B); however, the estimate also included the possibility of no between-group difference. No studies have reported quality of life between HBE and HBE combined with CBE. Only one study (27) reported the patient-reported pain and physical disability at long-term, and reduction in pain in HBE combined with CBE (*n* = 103) was noted to be statistically significant compared with HBE alone (*n* = 79).

Three studies (25, 43, 44) were not included in the meta-analysis due to inconsistent interventions. Two studies showed no difference in short-term pain and physical disability (43, 44) and long-term pain and physical disability (44) in HBE compared with HBE combined with CBE. One study (25) showed poorer benefits for HBE than HBE combined with CBE in short-term pain and physical disability. Regular monitoring has been shown to augment the efficacy of HBE (45). The first 2 studies had regular remote monitoring in HBE (43, 44), whereas the latter one (25) had no monitoring, which may account for the inconsistent findings.

#### 3.6.2 Secondary outcomes

Only One study (26) reported no between-groups difference for balance function at short-term. No studies reported long-term performance-based outcome measures comparing HBE and HBE combined with CBE.

### 3.7 Additional analyzes

Subgroup analyzes were not performed owing to the limited number of studies for each outcome. Sensitivity analyzes were conducted to evaluate the robustness of our meta-analysis findings when more than two studies were included. These sensitivity analyzes did not alter any of the meta-analysis results, which suggests this finding is robust.

### 3.8 Role of the funding source

The funders played no role in the design, conduct, or reporting of this study.

## 4 Discussion

This systematic review provides moderate-quality evidence that HBE was as effective as CBE for pain, physical disability, and walking ability in the short term. Based on low-quality evidence, there were also similar effects between HBE and CBE for lower limb muscle strength in the short term. When comparing HBE and HBE combined with CBE, there was moderate-quality evidence showing that HBE was inferior to HBE combined with CBE in short-term pain and physical disability. However, these findings need to be interpreted with caution as the number of included studies and participants is low. Insufficient data prevented the determination of differences beyond the intervention period. These findings imply that incorporating home-based exercise could serve as a complementary approach to center-based exercise, or that there is a need for future enhancements in the clinical effectiveness of center-based exercise for knee osteoarthritis.

This is the first systematic review and meta-analysis to specifically investigate the effects between HBE and CBE for people with KOA, resulting in a challenge to compare with previous reviews. A systematic review (24) published in 2016 reported that HBE and other treatments had similar effects, but the conclusion is conflicting because of inconsistent comparisons. Through six RCTs with consistent comparisons, this review enhances the findings that HBE yields benefits similar to CBE for pain, physical disability, walking ability, and lower limb muscle strength in the short term. As only studies with identical exercise interventions in both the intervention and control groups were included in the meta-analyzes, this approach effectively eliminates any confounding effects resulting from differences in intervention location and contents. GRADE provides very low-to moderate-quality evidence that HBE is as effective as CBE. The main reason for the lower quality of evidence is the restricted number of studies and participants included. In comparing HBE to CBE, it was observed that nearly all HBE programs incorporated regular telephone monitoring or supervision, although the frequency of such monitoring was only minimal weekly. These findings suggest that minimal supervised HBE has significant potential as a viable alternative to CBE in future clinical practice.

The second aim of this review was to determine the efficacy of HBE alone compared with HBE combined with CBE for people with KOA. Meta-analyzes of 2 RCTs suggest that additional sessions of supervised CBE may enhance the efficacy of HBE in short-term pain and physical disability. This finding is consistent with a previous study (46) demonstrating greater effectiveness in pain and physical disability with more supervised sessions when compared to a non-exercise control. Nevertheless, insufficient evidence from two RCTs precludes the definitive conclusion that HBE combined with CBE is superior to HBE alone in this review. Additionally, all HBE programs were without any telephone monitoring or supervision, which probably underestimated the effectiveness of HBE (47). Further, high-quality RCTs are necessary to establish conclusive evidence regarding the comparative efficacy of HBE alone versus HBE combined with CBE.

Exercise is one of the treatments that clinicians can deliver using telerehabilitation, and remote information technology can greatly enhance the effectiveness of home-based exercise. However, HBE has typically been used as an active control group in previous studies when compared with CBE in people with KOA, few studies have utilized it as the primary intervention. When home-based exercise versus home-based exercise supplemented center-based rehabilitation, several studies (43, 44) showed that additional center-based sessions probably showed a little increased effect. This is congruent with the findings of two reviews (48, 49) in orthopedic rehabilitation, where they found that additional outpatient interventions were not superior to home-based rehabilitation.

HBE tends to be more effective when it is supported by information technology, such as regular telephone monitoring (38–41, 43, 44), and CBE seems to be more effective when it is group-based (39). Future studies should maximize the efficacy of home rehabilitation by information technology or behavior promotion techniques, and then compare it with evidence-based clinic-based rehabilitation. The KOA’s latest clinical practice guidelines, while with an emphasis on home-based exercise (6, 7, 50), fail to recommend an optimal supervision frequency. Hence, there is a need to explore whether the combination of home-based rehabilitation and clinic-based rehabilitation would be superior to either intervention alone, and investigate the dose–response relationship of supervision. We have identified several ongoing randomized trials attempting to address these questions (51–54).

### 4.1 Limitations

There are several limitations to this systematic review with meta-analysis worth noting. Fewer trials and a small sample size limited the precision of the findings and undermined the capacity to assess publication bias graphically or statistically. Additionally, although data were extracted and pooled based on the priority of outcome indicators as recommended (5, 33), the pooled effect was calculated using the SMD, which is less clinically meaningful than a mean difference. When comparing HBE and HBE combined with CBE, no definitive conclusions were reached due to the limited number of studies. Future studies need to ascertain the amount of CBE supplementation for HBE that will have optimal cost-effectiveness, providing essential guidance for future clinical practice regarding people with KOA. On the other hand, individual adherence is the core component of HBE (55, 56), yet less reported in studies of HBR for KOA patients. Only one trial (27) reported adherence included in this review, showing no statistical difference between HBE and CBE. Lastly, fewer studies reported quality of life, performance-based outcomes, and long-term outcomes, leading to controversial results. Nevertheless, as the first systematic review to examine the effectiveness of HBE versus CBE, we can still have an impact on current clinical practice and future clinical research. More high-quality studies comparing the efficacy of HBE and CBE (with or without HBE) are needed in the future to strengthen the findings of this review.

### 4.2 Clinical implication

Home-based exercise presents comparable benefits to center-based exercise in terms of patient-reported and performance-based outcomes among people with knee osteoarthritis. This highlights the importance of prioritizing the incorporation of home exercise regimens into clinical guidelines for this population. Home-based exercise can effectively mitigate pain and functional deterioration in people with knee osteoarthritis with less medical supervision, hence decreasing the national healthcare burden. Furthermore, concurrent application of home-based and center-based rehabilitation may lead to superior outcomes. However, given the limited number of studies on the matter, it remains uncertain what the optimal number of supervised outpatient rehabilitation sessions would represent the most cost-effective strategy for the management of knee osteoarthritis. Further clinical research is warranted to address this issue. Finally, future studies should also include health economic analyzes and larger sample sizes. This could allow more robust subgroup analyzes and explore which specific subgroups might benefit most from home-based exercise.

## 5 Conclusion

In conclusion, this review provides evidence that HBE is as effective as CBE for KOA on pain, physical disability, walking ability, and lower limb muscle strength in short-term follow-up. Furthermore, with limited evidence, the efficacy of HBE maybe be enhanced by combined with CBE in short-term pain and physical disability. This review comprehensively synthesizes the differential efficacy of HBE compared with CBE for KOA, and the findings indicate that HBE could potentially serve as a favorable substitute for CBE in clinical settings characterized by limited healthcare resources or geographical constraints.

## Data availability statement

The original contributions presented in the study are included in the article/Supplementary material, further inquiries can be directed to the corresponding authors.

## Author contributions

Z-YZ: Conceptualization, Methodology, Writing – original draft. LH: Conceptualization, Methodology, Writing – original draft. LT: Data curation, Formal analysis, Methodology, Writing – original draft. JY: Conceptualization, Writing – review & editing. MG: Data curation, Methodology, Writing – original draft. X-QW: Data curation, Investigation, Writing – original draft. J-JJ: Conceptualization, Supervision, Writing – review & editing. Z-LL: Conceptualization, Supervision, Writing – review & editing.
