# Supplementary material for: Home-based vs center-based exercise on patient-reported and performance-based outcomes for knee osteoarthritis: a systematic review with meta-analysis
Source: Front Public Health. 2024 Mar 14;12:1360824. doi: 10.3389/fpubh.2024.1360824 (PMC10973546; doi:10.3389/fpubh.2024.1360824)
Supplement: Supplementary file 1 [file Table_1.DOCX]

Supplementary Material

**Supplemental Appendix1. Example search strategy.**

The search strategy for PubMed was shown below. It was adapted for the other databases.

| **Pubmed**  ("osteoarthritis, knee"[MeSH Terms] OR (("osteoarthr*"[Title/Abstract] OR "arthrosis"[Title/Abstract] OR "OA"[Title/Abstract] OR "degenerative arthritis"[Title/Abstract]) AND ("knee"[MeSH Terms] OR "knee joint"[MeSH Terms] OR "knee*"[Title/Abstract]))) AND ("exercise"[MeSH Terms] OR "physical fitness"[MeSH Terms] OR "Physical Therapy Specialty"[MeSH Terms] OR "telerehabilitation"[MeSH Terms] OR ("exertion"[Title/Abstract] OR "exercis*"[Title/Abstract] OR "physical therapy"[Title/Abstract] OR "telerehab*"[Title/Abstract] OR "balanc*"[Title/Abstract] OR "strength*"[Title/Abstract] OR "kinesiotherap*"[Title/Abstract] OR "resist*"[Title/Abstract] OR "thai chi"[Title/Abstract] OR "walk*"[Title/Abstract] OR "train*"[Title/Abstract] OR "aerobic*"[Title/Abstract] OR "physical activit*"[Title/Abstract] OR "treadmill*"[Title/Abstract] OR "run"[Title/Abstract] OR "mobility"[Title/Abstract] OR "qi gong"[Title/Abstract] OR "self manag*"[Title/Abstract])) AND ("home"[All Fields] OR "tele*"[All Fields] OR "remote"[All Fields]) | **Adapted for:**  Cochrane, Embase,  Web of Science, and Scopus. |
| --- | --- |

**Supplemental Appendix 2. Hierarchy of Patient-reported Outcome Measures**

**Patient-reported pain:**

1. Global pain

2. Pain on walking

3. Western Ontario and McMaster Osteoarthritis Index (WOMAC) osteoarthritis pain subscore.

4. Composite pain scores other than WOMAC.

5. Pain on activities other than walking.

6. Pain at rest or pain during the night.

7. WOMAC global algofunctional score.

8. Lequesne Osteoarthritis Index global score.

9. Other algofunctional scale

**Patient-reported function:**

1. Global disability score.

2. Walking disability.

3.WOMAC disability subscore.

4.Composite disability scores other than WOMAC.

5.Disability other than walking.

6.WOMAC global scale.

7. Lequesne Osteoarthritis Index global score.

8.Other algofunctional scale.

**Patient-reported Quality of Life:**

1. Short Form (SF)-36, Mental Component Summary (MCS).

2. SF-12 MCS

3. EuroQoL.

4. Sickness Impact Profile (SIP)

5. Nottingham Health Profile (NHP)

6. Other quality of life scales.

**Supplemental Appendix 3. GRADE Downgrade Details:**

(1) Risk of bias: If 25% of participants were from trials at high overall risk of bias, we downgraded by 1 level, and if 50% of participants were from trials at high overall risk of bias, we downgraded by 2 levels.

(2) Inconsistency: Evidence of considerable substantial (I^2^ >50%) was downgraded by 1 level, and evidence of considerable inconsistency (I^2^ >75%) was downgraded by 2 levels.

(3) Imprecision: if the total sample size was <400, we downgraded by 1 level.

(4) Publication bias: There were insufficient trials to produce funnel plots. Instead, we assessed publication bias based on (A) risk of selective reporting of results and other evidence of publication bias including (B) industry sponsorship or reported conflicts of interest. If 50% of participants were from studies in which A and B existed, we downgraded by 1 level.

(5) Indirectness: the indirectness criterion did not apply because the eligibility criteria ensured a defined group with relevant outcomes.

**Supplemental Appendix 4. Studies Excluded After Full-Text Review**

16 for wrong inclusion criteria(1–16)

4 for wrong study design(17–20)

3 for incomplete data(21–23)

**Reference**

1. Tore NG, Oskay D, Haznedaroglu S. The quality of physiotherapy and rehabilitation program and the effect of telerehabilitation on patients with knee osteoarthritis. *Clinical rheumatology* (2022) doi: 10.1007/s10067-022-06417-3

2. Hunter DJ, Bowden JL, Hinman RS, Egerton T, Briggs AM, Bunker SJ, French SD, Pirotta M, Shrestha R, Schofield DJ, et al. Effectiveness of a new service delivery model for management of knee osteoarthritis in primary care: a cluster randomised controlled trial. *Arthritis care & research* (2022) doi: 10.1002/acr.25037

3. Dieter V, Haupt G, Janssen P, Krauss I. RANDOMIZED-CONTROLLED TRIAL TO EVALUATE A 12-WEEK APP- AND BRACE-ASSISTED EXERCISE INTERVENTION IN PATIENTS WITH KNEE OSTEOARTHRITIS. *Osteoarthritis Cartilage* (2022) 30:S401‐S402. doi: 10.1016/j.joca.2022.02.539

4. Bozgeyik S, Kinikli GI, Topal Y, Beydagi MG, Turhan E, Kilinç HE, Güney-Deniz H. Supervised exercises have superior effects compared to home-based exercises for patients with knee osteoarthritis following platelet-rich plasma injection. *Res Sports Med* (2022) doi: 10.1080/15438627.2022.2102920

5. Xiao CM, Li JJ, Kang Y, Zhuang YC. Follow-up of a Wuqinxi exercise at home programme to reduce pain and improve function for knee osteoarthritis in older people: a randomised controlled trial. *Age Ageing* (2021) 50:570–575. doi: 10.1093/ageing/afaa179

6. Gohir SA, Eek F, Kelly A, Abhishek A, Valdes AM. Effectiveness of Internet-Based Exercises Aimed at Treating Knee Osteoarthritis: The iBEAT-OA Randomized Clinical Trial. *JAMA Netw Open* (2021) 4:e210012. doi: 10.1001/jamanetworkopen.2021.0012

7. Khachian A, Seyedoshohadaei M, Haghani H, Amiri F. Effect of self-management program on outcome of adult knee osteoarthritis. *Int J Orthop Trauma* (2020) 39: doi: 10.1016/j.ijotn.2020.100797

8. Huang Z, Zhong X, Xie Z, Tianwang L. Op0156 Hpr the Feasibility and Effectiveness of Telemedicine for Knee Osteoarthritis in Disease Management: A Randomised Control Trial. *Ann Rheum Dis* (2019) 78:153–153. doi: 10.1136/annrheumdis-2019-eular.6474

9. Cagnin A, Choiniere M, Durand M, Mezghani N, Gaudreault N, Hagemeister N. Impact of a personalized home exercise program for knee osteoarthritis patients on 3d kinematics: a cluster randomized controlled trial. *Osteoarthritis Cartilage* (2019) 27:S34‐S35. doi: 10.1016/j.joca.2019.02.051

10. Yilmaz M, Sahin M, Algun C. Effectiveness of home based exercise program taught by physiotherapist on pain and function in knee osteoarthritis. *Osteoporosis international* (2018) 29:S452. doi: 10.1007/s00198-018-4465-1

11. Loew L, Brosseau L, Kenny GP, Durand-Bush N, Poitras S, De Angelis G, Wells GA. An evidence-based walking program among older people with knee osteoarthritis: the PEP (participant exercise preference) pilot randomized controlled trial. *Clin Rheumatol* (2017) 36:1607–1616. doi: 10.1007/s10067-017-3606-9

12. Kloek CJJ, Bossen D, De Bakker DH, Dekker J, Veenhof C. Blended intervention with reduced face-to-face contact and usual physiotherapy show similar effectiveness in patients with osteoarthritis: a randomized controlled trial. *Physiotherapy* (2016) 102:e146. doi: 10.1016/j.physio.2016.10.168

13. Hawkins K, Ghazi F. The addition of a supervised exercise class to a home exercise programme in the treatment of patients with knee osteoarthritis following corticosteroid injection: A pilot study. *Int Musculoskelet Med* (2012) 34:159–165. doi: 10.1179/1753615412Y.0000000008

14. Espanha MM, Lago R, Carvalho MJ, Rego AJ, Teles J. Effects of exercise program on pain, joint stiffness and physical function in elderly patients with knee osteoarthritis. Nursing-based versus home-based Exercise. *Osteoarthritis and Cartilage*. (2010). p. S25–S26 doi: 10.1016/S1063-4584(10)60067-2

15. Shelton ML, Lee JQ, Morris GS, Massey PR, Kendall DG, Munsell MF, Anderson KO, Simmonds MJ, Giralt SA. A randomized control trial of a supervised versus a self-directed exercise program for allogeneic stem cell transplant patients. *Psychooncology* (2009) 18:353–359. doi: 10.1002/pon.1505

16. Stitik TP, Blacksin MF, Stiskal DM, Kim JH, Foye PM, Schoenherr L, Choi E-S, Chen B, Saunders HJ, Nadler SF. Efficacy and safety of hyaluronan treatment in combination therapy with home exercise for knee osteoarthritis pain. *Arch Phys Med Rehabil* (2007) 88:135–141. doi: 10.1016/j.apmr.2006.11.006

17. Primeau CA, Birmingham TB, Thomas G, Olver T, Lorbergs AL, Moyer RM, Leitch KM, Giffin J. The feasibility and efficacy of a 12-week body re-composition and neuromuscular exercise program in patients with knee osteoarthritis. *Osteoarthritis Cartilage* (2019) 27:S497–S498. doi: 10.1016/j.joca.2019.02.556

18. Rogers M, Tamulevicius N, Semple S, Coetsee M, Curry B. Comparison of clinic-based versus home-based balance and agility training for the symptoms of knee osteoarthritis. *South African Sports Medicine Association* (2011) 23:

19. Konishi I, Tanabe N, Seki N, Suzuki H, Okamura T, Shinoda K, Hoshin E. Physiotherapy Program through Home Visits for Community-Dwelling Elderly Japanese Women with Mild Knee Pain. *Tohoku J Exp Med* (2009) 219:91–99. doi: 10.1620/tjem.219.91

20. Chamberlain MA, Care G, Harfield B. Physiotherapy in osteoarthrosis of the knees: A controlled trial of hospital versus home exercises. *Disabil Rehabil* (1982) 4:101–106. doi: 10.3109/09638288209166889

21. Pekesen Kurtça M, Baş Aslan U, Kuyucu E. HPR a comparison the effectiveness of wholebody vibration, progressive resistive exercise and home-based exercise in patients with knee osteoarthr it is. *Ann Rheum Dis* (2017) 76:1484. doi: 10.1136/annrheumdis-2017-eular.6854

22. Aslan UB, Kurtça MP, Koçyiǧit F, Koçyiǧit A, Kuyucu E. Effectiveness of supervised resistive exercise and homebased exercise training on lower limb muscle strength in patients with knee osteoarthritis: A long-term comparative study. *Osteoarthritis Cartilage* (2016) 24:S477–S478.

23. Alayli G, Kuru Ö, Bilgici A. The effects of aerobic exercise and home exercise on pain and disability in patients with knee osteoarthritis. *J Rheumatol Med Rehabil* (2007) 18:46–50.

**Supplemental Appendix 5.**


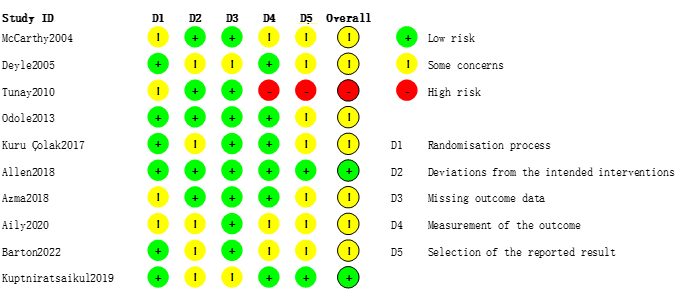


**Supplemental Appendix 6.**


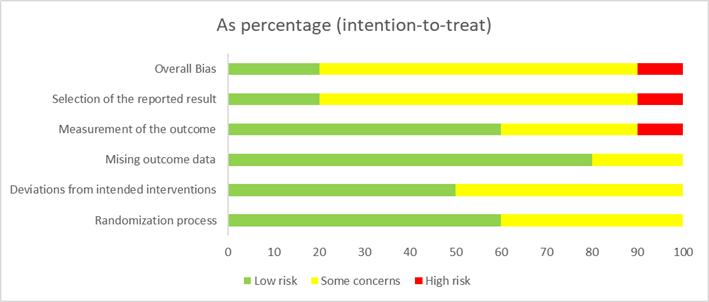


**Supplemental Appendix 7. GRADE for primary outcomes for HBE compared with CBE.**

| **HBE compared to CBE for Knee Osteoarthritis** | | | | | |
| --- | --- | --- | --- | --- | --- |
| Outcomes | **Anticipated absolute effects^*^** (95% CI) | | Relative effect (95% CI) | № of participants (studies) | Certainty of the evidence (GRADE)  Comments |
|  | **Risk with equivalent CBE** | **Risk with HBE** |  |  |  |
| Pain-short-term | - | SMD 0.22 higher (0.04 lower to 0.47 higher) | - | 270 (5RCTs) | ⨁⨁⨁◯ Moderate^a^ |
| Physical disability-short-term | - | SMD 0.17 higher (0.19 lower to 0.54 higher) | - | 114 (3 RCTs) | ⨁⨁⨁◯ Moderate^a^ |
| ***The risk in the intervention group** (and its 95% confidence interval) is based on the assumed risk in the comparison group and the **relative effect** of the intervention (and its 95% CI).  **CI:** confidence interval; **SMD:** standardised mean difference | | | | | |
| **GRADE Working Group grades of evidence** **High certainty:** we are very confident that the true effect lies close to that of the estimate of the effect. **Moderate certainty:** we are moderately confident in the effect estimate: the true effect is likely to be close to the estimate of the effect, but there is a possibility that it is substantially different. **Low certainty:** our confidence in the effect estimate is limited: the true effect may be substantially different from the estimate of the effect. **Very low certainty:** we have very little confidence in the effect estimate: the true effect is likely to be substantially different from the estimate of effect. | | | | | |

#### Explanations

1. Total sample size was <400

**Supplemental Appendix 8. GRADE for Secondary Outcomes for HBE compared with CBE.**

| **HBE compared to CBE for Knee Osteoarthritis** | | | | | |
| --- | --- | --- | --- | --- | --- |
| Outcomes | **Anticipated absolute effects^*^** (95% CI) | | Relative effect (95% CI) | № of participants (studies) | Certainty of the evidence (GRADE) |
|  | **Risk with equivalent CBE** | **Risk with HBE** |  |  |  |
| Walking ability-short-term | - | SMD 0.21 lower (0.64 lower to 0.22 higher) | - | 146 (3 RCTs) | ⨁⨁⨁◯ Moderate^a^ |
| Lower limb muscle strength-short-term | - | SMD 0.24 lower (0.88 lower to 0.41 higher) | - | 146 (3 RCTs) | ⨁◯◯◯ Low^a,b^ |
| ***The risk in the intervention group** (and its 95% confidence interval) is based on the assumed risk in the comparison group and the **relative effect** of the intervention (and its 95% CI).  **CI:** confidence interval; **SMD:** standardised mean difference | | | | | |
| **GRADE Working Group grades of evidence** **High certainty:** we are very confident that the true effect lies close to that of the estimate of the effect. **Moderate certainty:** we are moderately confident in the effect estimate: the true effect is likely to be close to the estimate of the effect, but there is a possibility that it is substantially different. **Low certainty:** our confidence in the effect estimate is limited: the true effect may be substantially different from the estimate of the effect. **Very low certainty:** we have very little confidence in the effect estimate: the true effect is likely to be substantially different from the estimate of effect. | | | | | |

#### Explanations

a. Total sample size was <400

b. Evidence of substantial considerable (I2 >50%)

**Supplemental Appendix 9. GRADE for primary Outcomes for HBE compared with HBE combined with CBE**

| **HBE compared to HBE combined with CBE for Knee Osteoarthritis** | | | | | |
| --- | --- | --- | --- | --- | --- |
| Outcomes | **Anticipated absolute effects^*^** (95% CI) | | Relative effect (95% CI) | № of participants (studies) | Certainty of the evidence (GRADE) |
|  | **Risk with HBE combined with CBE** | **Risk with HBE** |  |  |  |
| Pain-short-term | - | SMD 0.89 higher (0.60 higher to 1.17 higher) | - | 250 (2 RCTs) | ⨁◯◯◯ Moderate^a^ |
| Physical disability-short-term | - | SMD 0.25 higher (0 to 0.5 higher) | - | 250 (2 RCTs) | ⨁⨁⨁◯ Moderate^a^ |
| ***The risk in the intervention group** (and its 95% confidence interval) is based on the assumed risk in the comparison group and the **relative effect** of the intervention (and its 95% CI).  **CI:** confidence interval; **SMD:** standardised mean difference | | | | | |
| **GRADE Working Group grades of evidence** **High certainty:** we are very confident that the true effect lies close to that of the estimate of the effect. **Moderate certainty:** we are moderately confident in the effect estimate: the true effect is likely to be close to the estimate of the effect, but there is a possibility that it is substantially different. **Low certainty:** our confidence in the effect estimate is limited: the true effect may be substantially different from the estimate of the effect. **Very low certainty:** we have very little confidence in the effect estimate: the true effect is likely to be substantially different from the estimate of effect. | | | | | |

#### Explanations

a. Total sample size was <400
